# Effects of upper-molar distalization using clear aligners in combination with Class II elastics: a three-dimensional finite element analysis

**DOI:** 10.1186/s12903-022-02526-2

**Published:** 2022-12-01

**Authors:** Xulin Liu, Yuxun Cheng, Wen Qin, Shishu Fang, Wei Wang, Yanning Ma, Zuolin Jin

**Affiliations:** 1 Department of Orthodontics, School of Stomatology, State Key Laboratory of Military Stomatology & National Clinical Research Center for Oral Diseases & Shaanxi Clinical Research Center for Oral Diseases, Air Force Medical University, Xi’an, 710032 China; 2 Urumql DW Innovation InfoTech Co.Ltd, Xinjiang, 830000 China; 3 Shanxi Medical University School and Hospital of Stomatology, Taiyuan, 030001 China

**Keywords:** Molar distalization, Clear aligners, Class II elastics, Finite element analysis

## Abstract

**Introduction:**

The effects of upper-molar distalization using clear aligners in combination with Class II elastics for anchorage reinforcement have not been fully investigated yet. The objective of this study is to analyze the movement and stress of the whole dentition and further explore guidelines for the selection of traction methods.

**Methods:**

Three-dimensional (3D) finite element models are established to simulate the sequential molar distalization process, including the initial distalization of the 2^nd^ molar (Set I) and the initial distalization of the 1^st^ molar (Set II). Each group set features three models: a control model without Class II elastics (model A), Class II elastics attached to the tooth by buttons (model B), and Class II elastics attached to the aligner by precision cutting (model C). The 3D displacements, proclination angles, periodontal ligament (PDL) hydrostatic stress and alveolar bone von Mises stress in the anterior area are recorded.

**Results:**

In all of the models, the maxillary anterior teeth are labial and mesial proclined, whereas the distal moving molars exhibit distal buccal inclination with an extrusion tendency. With the combination of Class II elastics, the anchorage was effectively reinforced; model C demonstrates superior anchorage reinforcement with lower stress distribution in comparison with model B. The upper canines in model B present an extrusion tendency. Meanwhile, the mandibular dentition in models B and C experience undesired movement tendencies with little discrepancy from each other.

**Conclusions:**

Class II elastics are generally effective for anchorage reinforcement as the upper-molar distalization is performed with clear aligners. Class II elastics attached to an aligner by precision cutting is a superior alternative for maxillary anchorage control in cases that the proclination of upper incisors and extrusion of upper canines are unwanted.

**Supplementary Information:**

The online version contains supplementary material available at 10.1186/s12903-022-02526-2.

## Introduction

Over recent decades, an increasing number of patients have accepted clear aligner treatment (CAT) for both cosmetic and comfort reasons. With the advancement of biomechanics and material science, significant progress has been made in CAT; this has led to an improvement in therapeutic efficacy. A recent study showed that the overall mean accuracy of the Invisalign aligner was 50% [1, 2].

Upper-molar distalization is a typical Class II non-extraction treatment strategy that is used to acquire dental space, establish Class I molar relationships and obtain a normal overjet. During distalization, conventional orthodontic methods persistently generate undesirable outcomes such as molar extrusion and tipping [3]. Over recent years, CAT has been used to improve vertical dimension control, rendering this a superior alternative for the treatment of patients with hyperdivergent or open bites [4]. Upper molar distalization is associated with the highest predictability of 88% when a bodily movement of at least 1.5 mm was prescribed [1]. However, due to the fact that molar distalization produces a reciprocal force on the anterior teeth, anchorage loss is inevitable [5], which can represent a substantial cause of alveolar defects, such as dehiscence and fenestration in some patients with thin cortical plates in the anterior region [6].

Class II elastics are commonly employed in conventional fixed multibracket therapy (FMB) for anchorage reinforcement and molar correction during maxillary molar distalization. Despite their popularity, recent investigations have revealed that the adverse effects of Class II elastics outweigh the intended objectives, such as clockwise rotation of the occlusal plane and the mandible, and worsening smile esthetics due to greater exposure of the gums [7]. In clinical conditions, clear aligners, as an overlay device, may avoid the undesirable vertical movement of Class II elastics caused by coverage of the entire dentition with the biting force. Thus, the mutual reinforcement of clear aligners and Class II elastics can facilitate the anchorage reinforcement in conjunction with vertical control [8]. A multicenter retrospective study conducted by Ravera et al. [9] demonstrated that the combination of clear aligners with composite attachments and Class II elastics caused distalization of the maxillary first molars by 2.25 mm without remarkable tipping and vertical movements of the crown, and there was also good buccolingual control of the upper incisors with respect to the palatal plane. However, this evaluation was performed at the end of treatment rather than focusing on the immediate effect at the end of the distalization phase. Class II elastics exploited in the previous study were only utilized after the distalization of the molars. In such a complicated force system, the biomechanical analysis of the maxillary and mandibular dentition has not yet been fully examined. Additionally, there are no clinical guidelines for the selection of traction methods for Class II elastics, including the bonding of buttons directly onto the tooth surface and the precise cutting of clear aligners.

The finite element method (FEM) is a numerical engineering technique used to instantly calculate initial tooth movement after force loading. This method is widely used in biomechanical investigations to evaluate displacement and stress responses in a variety of applications. Over recent years, the FEM has proven to be an effective tool to simulate tooth displacement patterns in orthodontics. However, the previous FE-based models only involved a single dentition within their study purposes [10, 11].

As far as the upper-molar distalization is performed with clear aligners in combination with Class II elastics, the maxillary dentition is inherently associated with the mandibular dentition by elastics. Therefore, it is necessary to establish rational FE-based models involving the whole dentition for comprehensive analysis, and the process of sequential molar distalization in clinical practice should be taken into consideration [12]. This is the first attempt to biomechanically evaluate the effect of clear aligners in combination with Class II elastics on both the maxillary and mandibular dentitions during upper-molar sequential distalization by FEM and further provide reference guidelines for the selection of traction methods.

## Methods

### CBCT taking

Cone-beam computed tomography (CBCT) data (GE Healthcare, USA) was attained from a 24-year-old female patient with healthy craniofacial structure and full dentition with extracted third molars and a Class II occlusion, which was taken previously for treatment purposes. The patient provided signed and informed consent to participate in the study and the use of her CBCT data was approved by the Ethics Committee of Shanxi Medical University School in China (No. 2022SLL009). The CBCT scan was performed with full FoV, a centered rotation of 360°. The voltage and current were 100 kV, 4 mA, and the exposure timing was 15 s. The thickness of each CBCT slice was set equal to 0.15 mm. All in all, 668 horizontal slices were reconstructed.

### Modeling the original 3D volume from CBCT

**Fig. 1:**
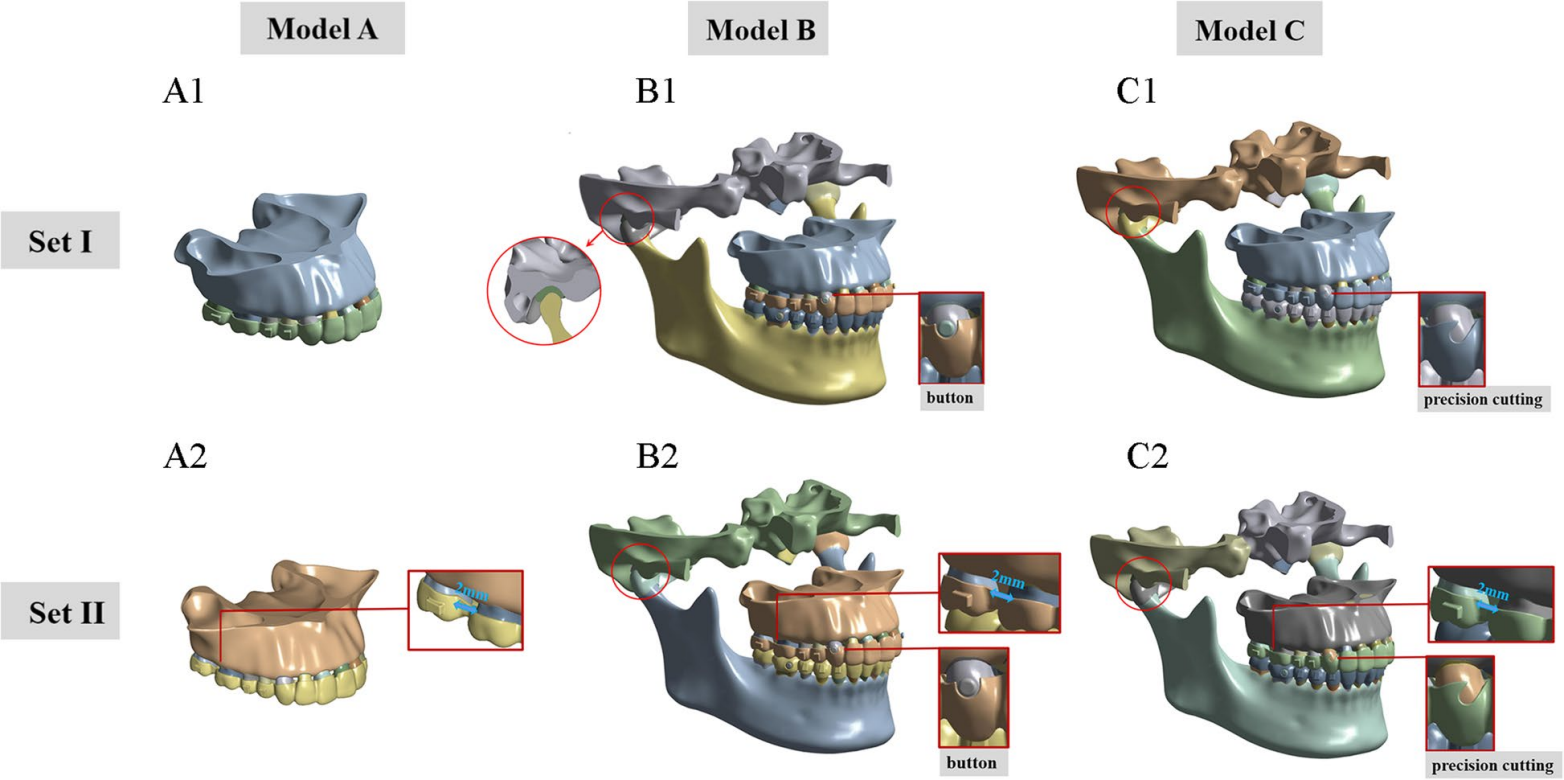
Finite element models. Two group sets including six sub-models were presented. Set I (A1, B1, and C1) represents the initial distalization of the 2^nd^ molar, Set II (A2, B2, and C2) simulated the initial distalization of the 1^st^ molar after 2 mm distalization of the 2^nd^ molar. Models A (A1, and A2) indicated maxillary models to simulate the upper-molar distalization using clear aligners without Class II elastics. Models B (B1, B2) were designed to simulate the upper-molar distalization using clear aligners in combination with Class II elastics by buttons. Models C (C1, and C2) were designed to simulate the upper-molar distalization using clear aligners in combination with Class II elastics by precision cutting. The red circles represent the temporal-mandibular joint

The CBCT data was imported into Mimics 20.0 software (Materialise Software, Leuven, Belgium). The mask layers of the maxilla and upper dentition, mandible and lower dentition, as well as temporal-mandibular joint (TMJ) were established by threshold operation. The Calculate 3D command was used to reconstruct the original 3D model. Geomagic Studio 2014 (Raindrop GEOMAGIC, North Carolina, USA) software was employed to optimize the original 3D models and create a surface model structure. The 3D mechanical drawing software NX 1911 (Siemens, Germany) was utilized to construct a preliminary model for periodontal ligament (PDL), alveolar bone, and attachments as previously described [13, 14]. PDL was modeled as a uniform layer by extending the outer surface of the tooth roots by 0.25 mm. The maxilla and mandible were moved inwards by 1.3 mm using the offset command and then cortical bone and cancellous bone models were established by Boolean subtraction operation instructions. The disc was modeled according to anatomical structure. Two layers of uniform thickness covering the condylar and temporal (0.41 mm) bone articular surfaces were produced to model the respective articular cartilages (Fig.1a) [15]. For retention purposes, vertical rectangular attachments(2 × 3 × 1 mm) were designed on the buccal surface of all the premolars and lower canine, and a horizontal rectangular attachment(3 × 2 × 1 mm) was designed on the upper 2^nd^ molars. The tooth crowns and attachments were extended outwards by 0.5 mm to simulate the clear aligner appliance.

### Creating sub-models from the original 3D model

All components were imported into ANSYS Workbench 2019 (Ansys, Pennsylvania, USA) to construct an appropriate 3D FE-based model for finite element analysis. we employed SOLID187, a 3D 10-node tetrahedral structural solid. The material properties were extracted from previous research works [14, 16–18] (Table 1). A linear viscoelastic model was employed for biomechanical modelling of the disc, which was implemented using a generalized Maxwell model via an optimized Prony series [15]. The other structures were assumed as linear, elastic, isotropic, and homogenous material. As shown in Fig. 1, two group sets including six sub-models were developed for simulations of the simplified process of the sequential molar distalization, the design inspiration of which came from previous studies [12, 19, 20]. Group set I is employed to simulate the initial distalization of the 2^nd^ molar, while Group set II is implemented to model the initial distalization of the 1^st^ molar after 2 mm distalization of the 2^nd^ molar. Model A (A1 and A2) represented maxillary models to simulate the upper-molar distalization by utilizing clear aligners without anchorage reinforcement. Model B (B1 and B2) was designed to simulate the upper-molar distalization by employing clear aligners in combination with Class II elastics by buttons. The buttons (diameter of bottom surface 3 mm, height 1 mm) were designed on the maxillary canine and the mandibular 1^st^ molar with corresponding parts of the clear aligner cut off. The interfaces of buttons and tooth surfaces were bonded. Model C (C1 and C2) was designed to simulate the upper-molar distalization using clear aligners in combination with Class II elastics by precision cutting. The precision cutting was designed on the maxillary canine by removing some parts of the appliance. The buttons and precision cutting were designed according to the clinical situation.

**Table 1:** Material properties

Material	Young’s modulus (MPa)	Poisson’s ratio
Tooth [14, 16, 18]	1.96 × 10^4^	0.3
PDL [14, 16, 18]	6.9 × 10^− 1^	0.45
Cortical bone [14, 16, 18]	1.37 × 10^4^	0.26
Cancellous bone [14, 16, 18]	1.37 × 10^3^	0.3
Clear Aligner [14, 18]	5.28 × 10^2^	0.36
Attachments [14, 16, 18]	1.25 × 10^4^	0.36
Button [17]	1.14 × 10^5^	0.35
Condylar cartilage [15]	8 × 10^− 1^	0.3
Temporal cartilage [15]	1.5	0.3
Disc [15]	1.8 × 10^− 1^	0.4

### The boundary and contact conditions of the sub-models

**Fig. 2:**
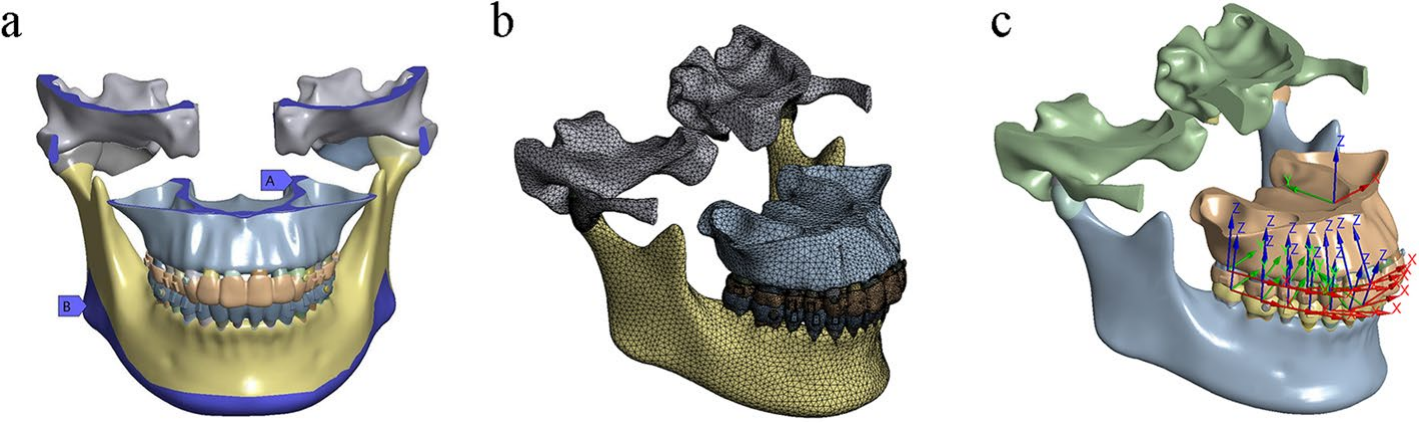
The boundary conditions, mesh figure, and coordinate systems. **a** The boundary conditions. Label A, the movement of temporal and maxilla bones were restricted for all degrees of freedom of the nodes at its superior region; Label B, the mandibular was also fixed at the lower margin of mandibular body. **b** A figure showing the mesh. c. Image showing the global and local coordinate systems

In regard to the boundary conditions (Fig. 2a), the movement of temporal and maxilla bone was restricted for all degrees of freedom of the nodes at its superior region, and the mandibular was fixed at the lower margin of the mandible. The bonding contact was set for interfaces of spongious-cortical bone, cortical bone-PDL, PDL-tooth, and tooth-attachment. Such a bonding did not allow any movement between contact faces. Further, the connections between the adjacent teeth were assumed to be no separation from their interfaces; however, small amounts of frictionless sliding are allowed to occur along the contact faces. A friction-based condition was established in the contact interfaces between the aligner and the tooth crown surface and attachments with a friction coefficient of 0.2 [16]. Between the articular cartilages and disc, surface-to-surface contacts were utilized with a friction coefficient of 0.001 [21]. Additionally, non-contact was considered between the maxillary and mandibular dentition.

### Loading

The FEA simulation was performed based on static loading. The step distance for molar distalization was set as 0.25 mm displacement along the y (+) axis. In group set I, the 2^nd^ molar was moved distally by 0.25 mm to generate a clear aligner under a loading condition; the loading force was then applied by the mismatch between the aligner and the initial dentition. In group set II, the 2^nd^ molar was moved 2 mm distally to a target position; then, the 1^st^ molar was moved 0.25 mm distally to generate a clear aligner under loading conditions [18]. In models B and C, a slight force of magnitude 120 g was also applied using a spring to simulate the force produced by Class II elastics which was explored through preliminary experiments [19].^.^

### Outcomes

The FE mesh was divided by the discretization process(Fig. 2b), nodes and linear elements of each sub-models are shown in Table 2 . We established two coordinate systems for reference (Fig. 2c). The global system is defined for the whole dentition, the *x*-axis represented the coronal plane (+ left, −right), the *y*-axis denoted the sagittal plane (+ posterior, −anterior), and the z-axis signified the vertical plane (+ superior, −inferior). The local coordination system was defined for each tooth as follows: the *x*-axis (+mesial, −distal), the *y*-axis (+lingual, −buccal), and the positive direction on the *z*-axis is represented by the apex of the maxillary teeth and the incisor/occlusal of the lower teeth. The initial movement of the whole dentition and the individual teeth in three directions were recorded. The proclination angle was measured by the intersection angle of the long axis of the tooth at the initial and final positions [22]. The PDL hydrostatic stress and von Mises stress of the alveolar bone in the anterior area were analyzed via the FEM.

**Table 2:** Nodes and elements

	A1	A2	B1	B2	C1	C2
Nodes	694,964	704,124	1,627,668	1,636,661	1,625,802	1,635,227
Elements	391,609	397,830	922,697	928,850	921,643	928,111

## Results

### Three-dimensional movement of the whole dentition

**Fig. 3:**
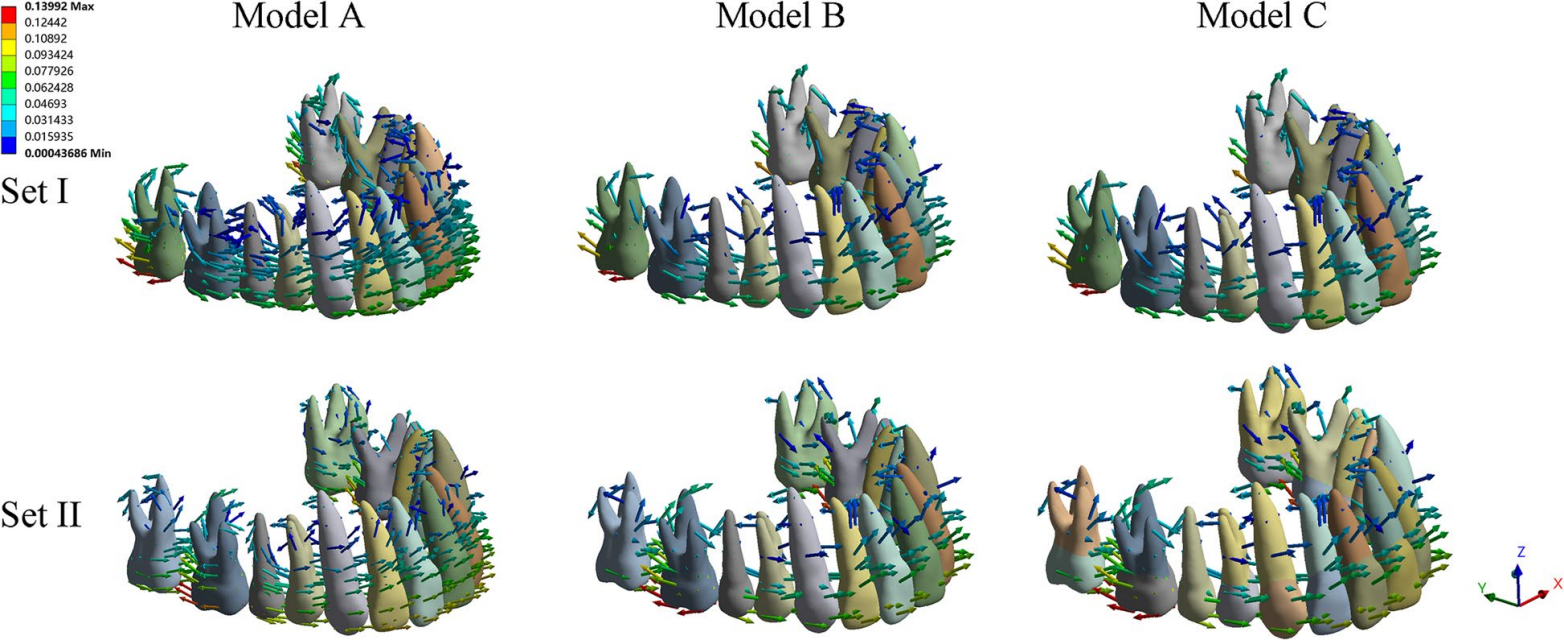
The movement tendency of the maxillary dentition. The coordinate system was based on the entire dentition (global coordinate system). Set I, initial distalization of the second molar; Set II, initial distalization of the first molar. Model A, control model without anchorage reinforcement; Model B, Class II elastics attached to the tooth by buttons; Model C, Class II elastics attached to the aligner by precision cutting. As shown by the arrows, with the distalization of the upper molars, the anterior and posterior teeth move in opposite directions due to the exerted reciprocal force. The *x*-axis represented the coronal plane (+ left, −right), the *y*-axis represented the sagittal plane (+ posterior, −anterior), and the *z*-axis represented the vertical plane (+ superior, −inferior)

**Fig. 4:**
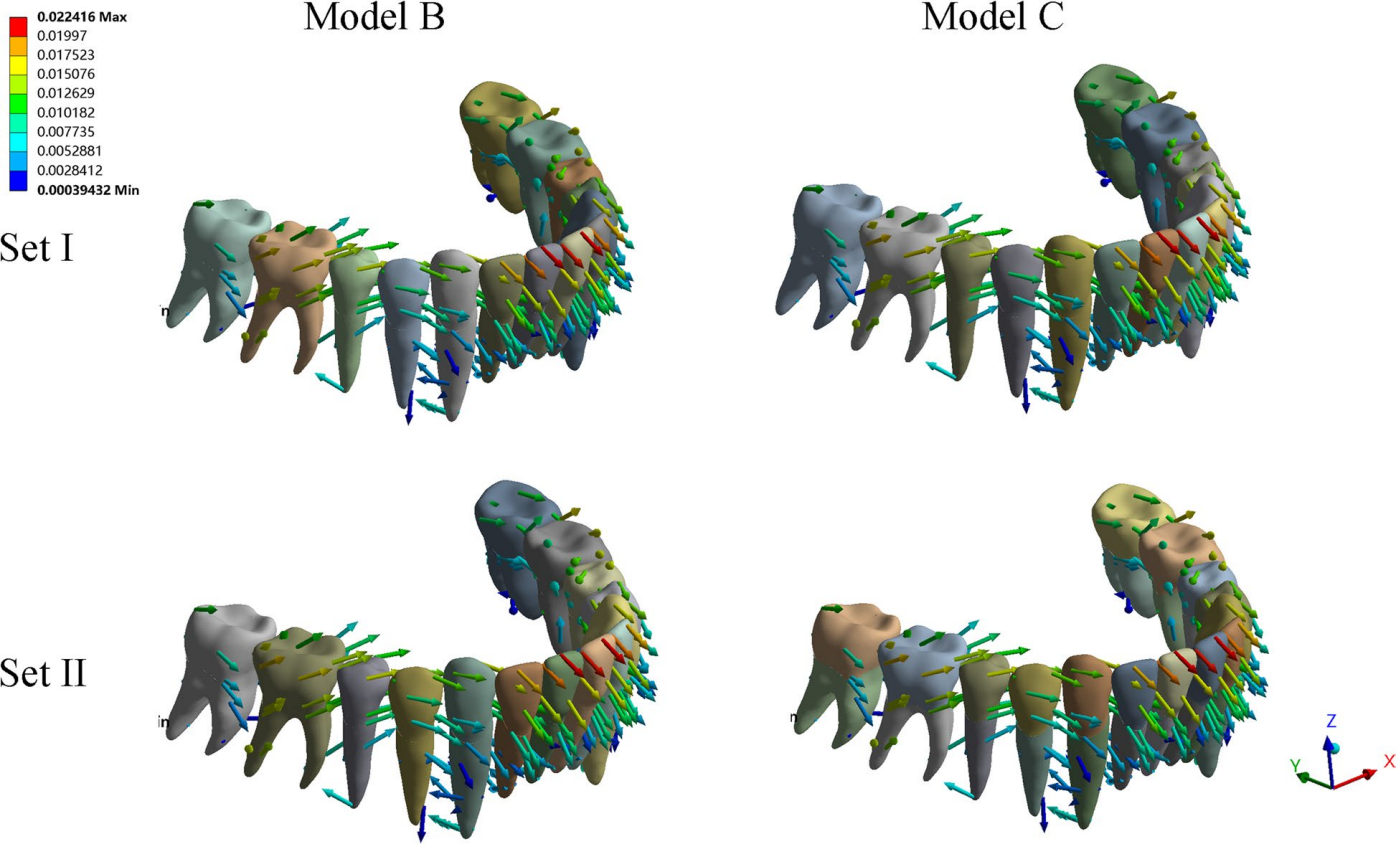
The movement tendency of the mandibular dentition in models with Class II elastics. The coordinate system was specified based on the entire dentition (global coordinate system). As shown by the arrows, with the combination of Class II elastic, the mandibular dentition of models B and C moved forward with a little extrusion tendency of molars and intrusion of anterior teeth

**Fig. 5:**
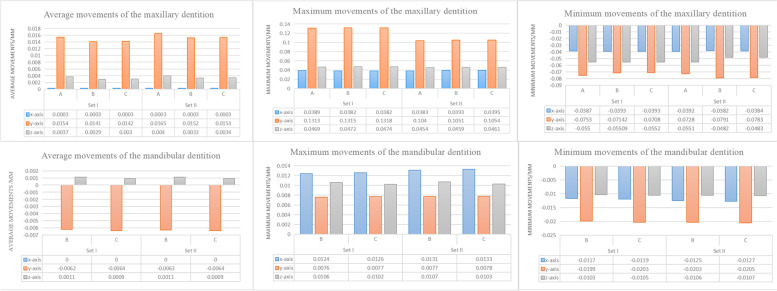
Bar charts of three-dimensional displacement for the maxillary and mandibular dentition (in mm). The coordinate system was considered based on the entire dentition (global coordinate system)

The movement of the whole dentition was recorded according to the global coordinate system. The dentition movement was almost in the sagittal plane (*y*-axis). The maxillary dentition in all the models moved backward with a little intrusion tendency, in which the anterior and posterior teeth move in opposite directions due to the reciprocal force (Fig. 3). The mandibular dentition of models B and C moved forward with extrusion tendency of molars and intrusion of anterior teeth (Fig. 4). There was little discrepancy between the results of model B and those of Model C according to the average, maximum and minimum value of the total deformation. The movement on *y*-axis of model A was the highest regarding average value(Fig. 5).

### Three-dimensional displacement of the anterior teeth

**Fig. 6:**
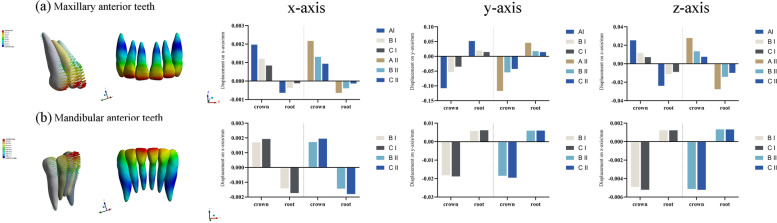
Three-dimensional displacement of the maxillary and mandibular anterior anchorage units. The vector diagrams and color maps showed initial patterns of movement. The anterior anchorage units exhibited labial and mesial inclination with a rotation center at the intersection of the apical and middle thirds of the roots for the maxillary incisors and the apical third of the roots for the mandibular incisors. The histograms show total displacement of the maxillary and mandibular anterior teeth displayed as crown and root displacement respectively (in mm). AI, model A1; AII, model A2; BI, model B1; BII, model B2; CI, model C1; CII, model C2. The coordinate system was centered on each tooth (local coordinate system); the positive value for the x-axis represents the mesial surface of the teeth, the positive value for y-axis represents the palatal surface of the teeth, and the z-axis represents a positive direction towards the apex of the maxillary teeth and the incisor/occlusal of lower teeth

As an anchorage unit, the maxillary anterior teeth exhibited mesial and labial proclination with a rotation center at the intersection of the apical and middle thirds of the roots in all of the models (Fig. 6a). In line with the force direction, the movement direction of the anterior teeth was approximately parallel to the *y*-axis based on the local coordinate system such that the crown displacement was larger than the root displacement for each tooth. With the initial distalization of the 1^st^ molar in group set II, the displacement tendency of the anterior teeth increased, thus indicating increased loss of anchorage. As the Class II elastics were used for anchorage enhancement, the uncontrolled movement of the anterior teeth decreased in models B and C compared with model A. As shown in supplementary file 1, the central incisor was flared to a relatively larger extent among the anterior teeth. Model C demonstrated superior anterior anchorage control compared with model B (y-axis displacement for set I: model A, − 0.1004; model B, − 0.0869; model C, − 0.0654; set II: model A, − 0.1113; model B, − 0.0935; model C, − 0.0718). The maxillary canine in model B exhibited rotation tendency with extrusion (Set I: *x*-axis, 0.0382, *y*-axis,-0.0295, *z*-axis,-0.0144; Set II: *x*-axis, 0.0406, *y*-axis,-0.0292, *z*-axis, − 0.0166). Notably, the mandibular anterior teeth in models B and C with Class II elastics exhibit mesial and labial proclination tendency with a rotation center at the apical third of the roots (see Fig. 6b). There was a trivial discrepancy between different models regarding the displacement values of the mandibular anterior teeth (supplementary file 2). The proclination angle of the incisors was measured and magnified fifty times for observation (Table 3). The range of the proclination angle was obtained as 1.64°- 4.25° for the maxillary central incisor, 1.50°- 3.90°for the maxillary lateral incisor, 0.85°- 0.97°for the mandibular central incisor, and 0.87°- 1.03° for the mandibular lateral incisor.

**Table 3:** Proclination angle of the incisors (50 x magnification)

Tooth	Model A		Model B		Model C	
	Set I	Set II	Set I	Set II	Set I	Set II
Maxillary central incisor	4.03°	4.25°	1.64°	1.81°	1.92°	2.13°
Maxillary lateral incisor	3.75°	3.90°	1.50°	1.73°	1.84°	2.05°
Mandibular central incisor	/	/	0.85°	0.89°	0.93°	0.97°
Mandibular lateral incisor	/	/	0.87°	0.92°	0.96°	1.03°

### Three-dimensional displacement of the molars

**Fig. 7:**
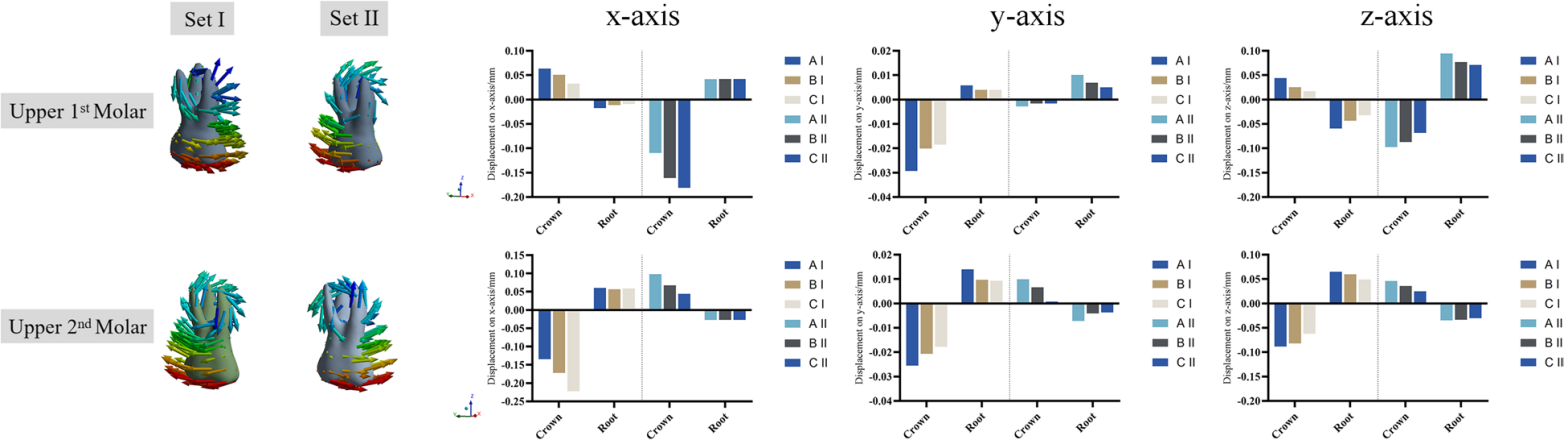
The movement tendency of the upper molars. The coordinate system was centered on each tooth (local coordinate system). In group set I, the 2^nd^ molar demonstrated distal and buccal inclination with extrusion tendency, while the 1^st^ molar presented mesial and buccal inclination with intrusion tendency. In group set II, the 1^st^ molar showed distal and buccal inclination with extrusion tendency, while the 2^nd^ molar demonstrated a tendency for mesial and palatal inclination with little intrusion

The direction of movement for the posterior teeth was mainly along the x-axis. In the control group, the 2nd molar in group set I showed distal and buccal inclination with extrusion tendency, while the 1^st^ molar showed mesial and buccal inclination with intrusion tendency (Fig.7). The 2^nd^ molar in group set II demonstrated a tendency for mesial and palatal inclination with little intrusion; this could be inferred as a relapse due to the reciprocal force of the 1^st^ molar distalization. With the combined use of Class II elastics for anchorage enhancement, the intended movement of the molars increased in the distal direction (Set I of the 2^nd^ molar on the x-axis: model A, − 0.1347; model B, − 0.1722; model C, − 0.2223), (Set II of the 1^st^ molar on the x-axis: model A, − 0.1097; model B, − 0.1611; model C, − 0.1811) (supplementary file 1). Furthermore, reduced displacement tendency was observed with regard to the relapse of the 2^nd^ molar in group set II (model A, 0.0979; model B, 0.0673; model C, 0.0442). Undesired movement tendency was decreased but not eliminated by anchorage enhancement with Class II elastics. As compared to model B, model C presented with an improved enhancement for the intended movement of molars in the distal direction and reduced relapse tendency of the 2^nd^ molar. When Class II elastics were used, the lower molars demonstrated mesial movement tendency with a tendency for extrusion and lingual movement (supplementary file 2).

### PDL hydrostatic stress and alveolar bone von Mises stress in the anterior area

**Fig. 8:**
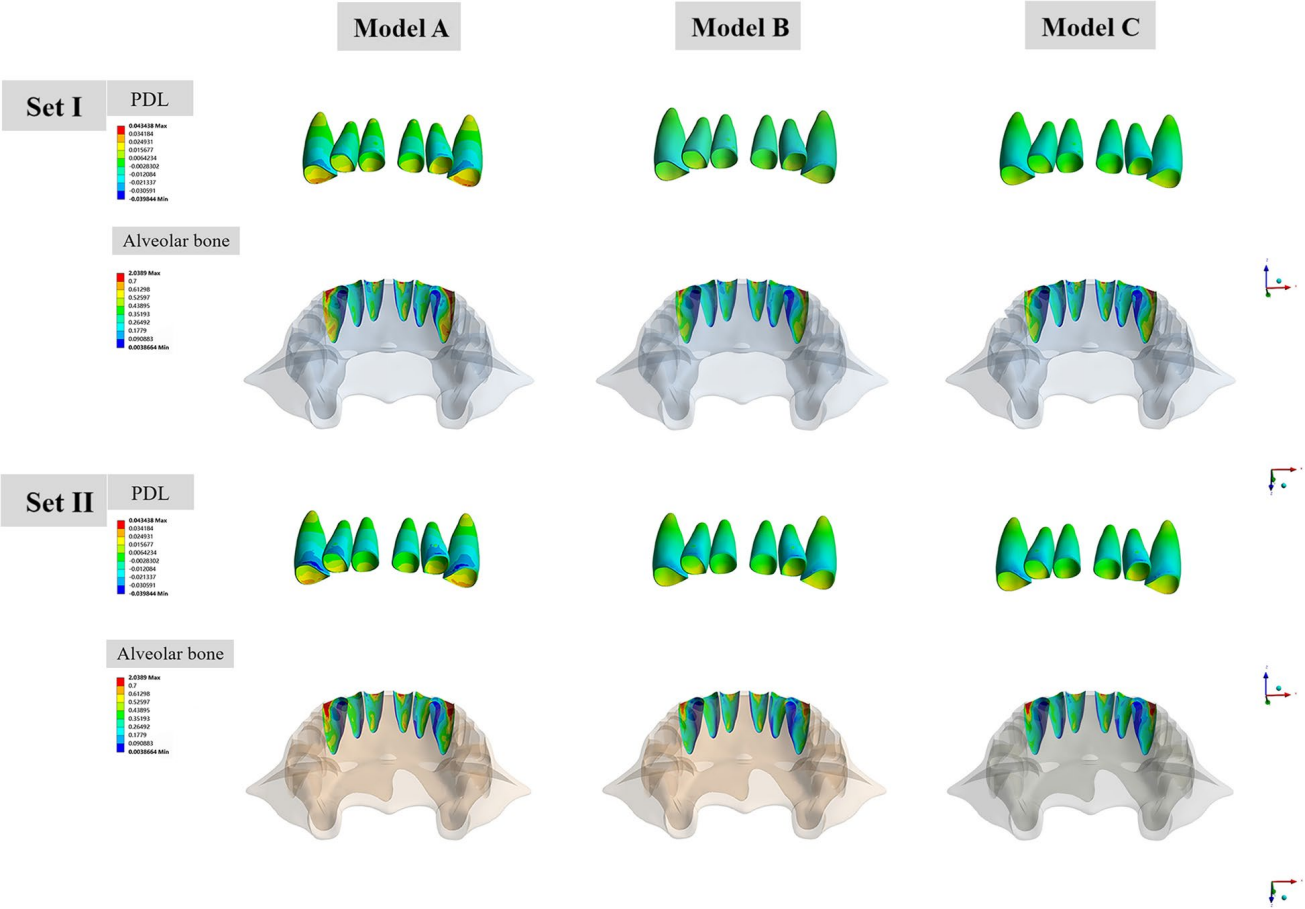
PDL hydrostatic stress and the von Mises stress of alveolar bone in maxillary anterior area (MPa). The front side represents buccal. For each sub-image, from middle to lateral are central incisors, lateral incisors, and canines. Positive values indicate tensile stresses for PDL hydrostatic stresses, while negative values represent compressive pressures. In all of the six models, the highest compressive stress of the PDL was concentrated on the labial cervical region and apex for upper incisors, and on the mesio-buccal cervical and apex area for upper canines. The stresses of alveolar bone were mostly distributed on the labial surface and concentrated on the cervical and apex region

**Fig. 9:**
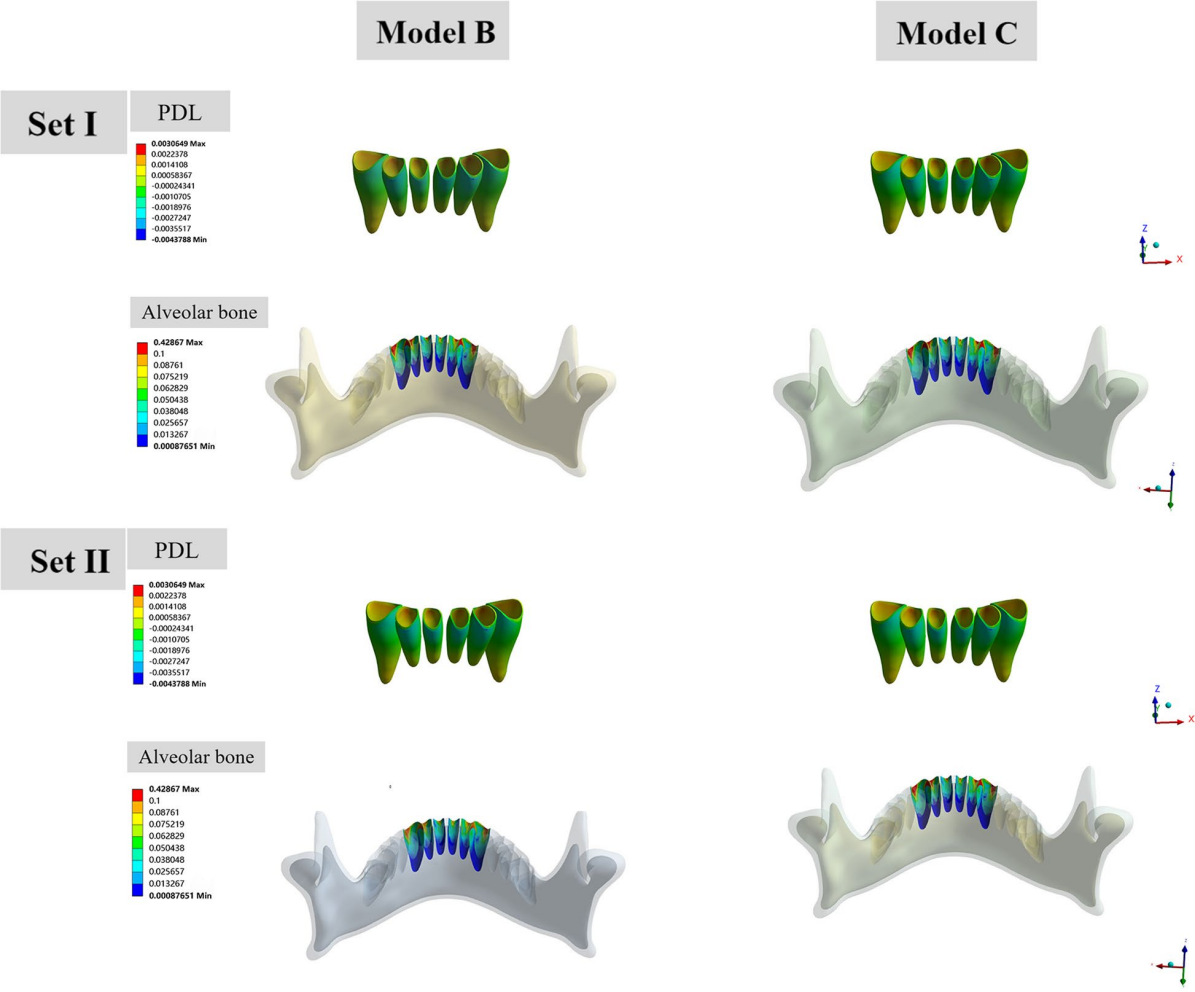
PDL hydrostatic stress and the von Mises stress of alveolar bone in mandibular anterior area (MPa). The front side represents buccal. For each sub-image, from middle to lateral are central incisors, lateral incisors, and canines. Positive values indicate tensile stresses for PDL hydrostatic stresses, while negative values represent compressive pressures. The stress of PDL and alveolar bone was concentrated on the labial cervical region for incisors, and on mesio-buccal cervical region for canine

**Fig. 10:**
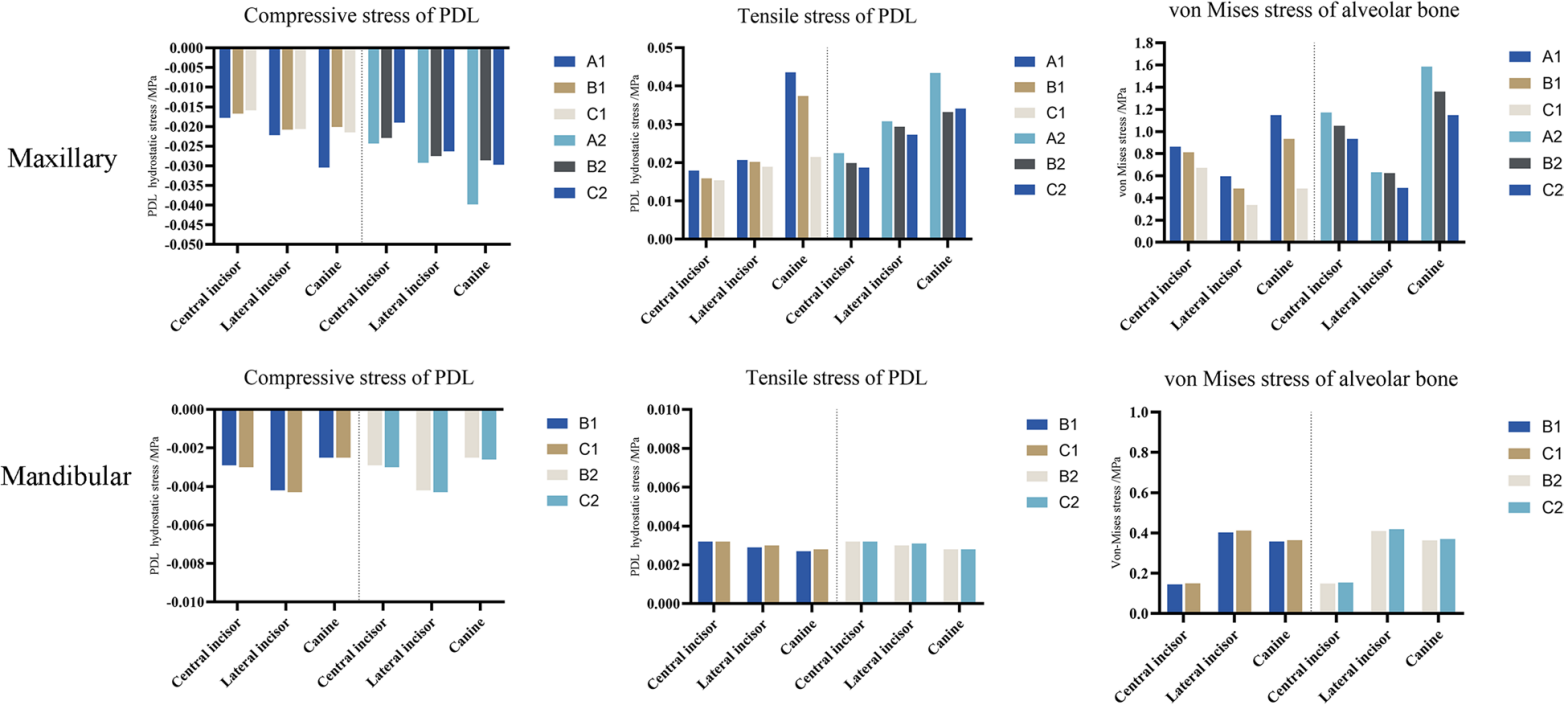
Bar charts of PDL hydrostatic stress and von Mises stress of alveolar bone in maxillary and mandibular anterior area. Positive values indicate tensile stresses for PDL hydrostatic stresses, while negative values represent compressive pressures

In all of the six models, the highest compressive stress of the PDL was concentrated on the labial cervical region and apex for upper incisors, while the highest tensile stress was distributed on the lingual cervical region (Fig. 8). For upper canines, the highest compressive stress was concentrated on the mesio-buccal cervical and apex area. The highest compressive stresses of maxillary anterior in all models were all above − 0.0047 MPa (in the interval of - 0.0389 MPa to - 0.0159 MPa), which is a threshold for a significant increase of the risk of external root resorption [23, 24 ]. model A presented the highest compressive stress value of the central incisor (Set I, - 0.0178 MPa, Set II, - 0.0243 MPa), lateral incisor (Set I, - 0.0222 MPa, - 0.0292 MPa), and canine (Set I, - 0.0304 MPa; Set II, − 0.0398 MPa). PDL hydrostatic stress was more evenly distributed in Model B and C with relatively lower compressive and tensile stress values. The minimum PDL hydrostatic stress was detected in Model C among all models (Fig. 10). The stresses of the alveolar bone were mostly distributed on the labial surface and concentrated on the cervical and apex region. Model A underwent the highest stress while the lowest stress was observed in Model C. The value of stress in group set II was relatively larger than that of group set I with similar stress distribution in the anterior area for each model. For the mandibular anterior area, small discrepancies were reported between the hydrostatic stress and alveolar bone von Mises stress of Models B and those of Model C (Fig. 9). The stress of PDL and alveolar bone was concentrated on the labial cervical region for incisors, and on mesio-buccal cervical region for canine. The highest compressive stress of the PDL in mandibular anterior area was all below the threshold level, ranging from - 0.0043 MPa to - 0.0025 MPa (Fig. 10).

## Discussion

CAT is known to exert distinct advantages for molar distalization. In a previous study, anterior flaring of teeth was reported in almost every patient to differing extents in patients who did not use auxiliaries other than composite attachments during the molar distalization process [25]. Class II elastics are extensively employed in FMB for anchorage reinforcement in the treatment of Class II malocclusion. However, the treatment with clear aligners and intermaxillary elastics is unexplored in many aspects. The previous clinical literature analyzing this treatment method in molar distalization is prevalently made of several retrospective studies and case reports [8, 26]. Several FE-based models about molar distalization with clear aligners only involved a single teeth or a single dentition, so that little attention was paid to the mandibular dentition. For instance, Rossini G, et al. [11] assessed the force system resulting in the upper arch during second maxillary molar distalization with clear aligners and variable attachment settings. Their FE analysis only involved a maxillary dentition without auxiliaries for anchorage reinforcement, and the sequential molar distalization process was not considered. Ayidaga C, et al. [13] analyzed the effect of different attachment configurations on the efficacy of bodily movement of the upper maxillary molar. Their simulation was limited to a single tooth, and the stress and movement analysis were only in the sagittal plane. Thus, further evidence is required for the specific effects of clear aligners in combination with Class II elastics on the entire dentition.

To the best of our knowledge, this is the first biomechanical study to establish comprehensive FE-based models involving both maxillary and mandibular dentition to simulate the sequential distalization of the upper molars using clear aligners in combination with Class II elastics. Uncontrolled tipping movement was observed for the entire dentition. Biomechanically, bodily movement and torque are the most demanding movements to achieve since plain aligners cannot establish the force required without modifications [27]. In such cases, the point of force application is passed above the resistance center of the teeth to produce a moment of rotation. Traditionally, the center of rotation was estimated by moment to force (M/F) ratios to predict the pattern of root movement, translation, or tipping around the apex [28]. In the present study, the rotation centers of the incisors were demonstrated visually by generating color maps that were roughly situated at the intersection of the apical and middle thirds of the roots for maxillary anterior teeth and at the apical third of the roots for mandibular anterior teeth. Consequently, the stresses on the PDL and alveolar bone were mostly concentrated on the cervical region of the labial surface. It is suggested that if the PDL hydrostatic pressure exceeds the capillary pressure in the area, the vessels will collapse and blood flow to that area will be impaired, increasing the risk of root resorption. As reported by previous investigations [23, 24], the threshold for capillary pressure in the PDL is estimated to be − 0.0047 MPa, which represents a substantial growth of the risk of external root resorption. Although the incidence is lower with CAT than with fixed appliances, root resorption cannot be avoided, particularly for maxillary and mandibular incisors [29]. In our experimental simulations, the highest compressive stresses of maxillary anterior PDL in all models were all concentrated on the labial cervical region and apex for upper incisors as well as mesio-buccal cervical and apex area of canines with values above − 0.0047 MPa, indicating a high risk of root resorption. With the class II elastics, models B and C have a relatively lower root resorption risk with more evenly distributed and relatively lower PDL hydrostatic stress value compared to model A. However, the root resorption risk could not be eliminated with the anchorage reinforcement of Class II elastics. As for the mandibular anterior, the highest compressive stress of the PDL was below the threshold level in all models, indicating a low risk of root resorption whether the Class II elastics were used or not. Additionally, the corresponding higher stress on the labial alveolar crest of the anterior area can lead to higher risks of bone defects, such as bone fenestration and dehiscence, which are frequently encountered in clinical practice, particularly with regard to the incisors [30]. By utilizing Class II elastics for anchorage reinforcement, proclination of anterior teeth has been controlled effectively with lower stress in models B and C, which may minimize the risks of root resorption and bone defects. Additionally, Class II elastics attached directly to the aligner by precision cutting result in superior anterior anchorage control when compared with buttons. Class II elastics applied to the tooth surface by buttons would cause more extrusion tendency with rotation of the specific canine compared with that applied to the aligner body. Under the conditions of Class II elastics, the mandibular anterior teeth also experienced undesirable labial movement, this was consistent with a recent retrospective study [26]. Nevertheless, the highest stress of the PDL in the mandibular anterior area was within the safe range with little discrepancy between models B and C. Comparing two group sets in our study, anterior anchorage loss grew by the initial distalization of the 1^st^ molar. It could be assumed that with the reduction in the distance between the anterior anchorage and the distalized molar, the force required magnifies, thus resulting in increased loss of anchorage. Therefore, enhanced protection of the anterior anchorage may be expected during the process of sequential distalization. In our observation, the proclination angles was measured and magnified according to clinical situation, in which the whole process of molar distalization was about 50 steps. Therefore, an optimized torque design can be proposed to facilitate the management of anchorage control [31].

With regards to the molars, the 2^nd^ molar exhibited a tendency for mesial and palatal inclination during the initial distalization of the 1^st^ molar. This relapse phenomenon could be attributed to the mesial reciprocal force produced by the distal displacement of the 1^st^ molar and the stress produced by the distal end of the aligner. This might explain why the maxillary 1^st^ molar exhibited relatively higher efficiency than the maxillary 2^nd^ molar during molar distalization [25]. In the present study, we found that Class II elastics reduced the relapse tendency of the 2^nd^ molar. Furthermore, attachment to the aligner by precision cutting demonstrated superior anchorage control which loaded and transmitted the anchorage force directly by the aligner. The attachment of Class II elastics to the teeth by buttons, however, loaded force onto the canines, so that the anchorage force was communicated primarily by the squeezing force between the neighboring teeth which was weakened by the gap between the teeth during transmission. Regardless of the traction method selected, some degree of anchorage loss was unavoidable, necessitating the use of an optimized attachment design to facilitate anchorage control.

Based on past research works and our current observations, the combined use of Class II elastics during maxillary molar distalization with aligners might effectively reinforce anchorage. From our biomechanical analysis, precision cutting might be a superior alternative when better anchorage control for both anterior teeth and molars is required, as well as the extrusion of upper canines are unwanted. For instance, Class II malocclusion division 1 with a deep overjet, which is often associated with labial inclined incisors and thin cortical bone in the maxillary anterior region, proclination of upper incisors is undesired when molars are designed to be distalized for a considerable distance. For another, hyperdivergent patients are not appropriate for extrusion of upper canines, which could cause the occlusal plane to incline and the Spee curve to deepen. However, in some cases, such as Class II division 2 malocclusion with a deep bite and retroclined incisors, the proclination and extrusion of anterior teeth are considered as a desired movement. Class II elastics attached by buttons would be more suitable. No matter what traction method is selected, mandibular anterior teeth will encounter proclination and intrusion to some extent, which is an underlying risk for patients with thin cortical plats in the mandibular anterior region. In the vertical direction, the extrusion tendency of the molars when moving distally, and that of the lower molars may cause the occlusal plane to incline and the Spee curve to deepen. However, the model was built with free space regardless of biting force on the aligners as an overlay appliance, which may prevent extrusion of the posterior teeth. For both traditional fixed multibracket therapy and clear aligner, the displacement patterns of the teeth are functions of the relationship between the center of resistance and the line of force. However, along a continuous archwire, a single distalizing force at the archwire level induced lingual inclination of the anterior segment due to the rigid connection between the segments [32]. In contrast, while clear aligners transmit force through appliance deformation, anterior teeth shifted in the opposite direction due to the reciprocal force created by molar distalization.

Nevertheless, as a biomechanical research tool, the FEM has intrinsic limits. In this study, the material properties of the subjects were simplifed based on the hypotheses derived from the average properties. The thickness of the periodontal ligament was assumed to be uniform, whereas in reality, it has an hourglass shape with the narrowest zone at the mid-root level [33]. As reported by Hohmann et al. [34], it is challenging to reconstruct PDL accurately from 3D image data obtained in vivo with its small dimensions. But they also proposed that for low loads as applied during typical orthodontic treatment, no noticeable discrepancies were found between the results generated with and without taking into account the geometric nonlinearities. Although the staging, loading methods, and traction methods were all designed in accordance with clinical practice and current studies, the efficacy of clear aligners in clinical practice is lower than predicted, owing to the materials of aligners and the manufacturing process. Clear aligners are made of thermoplastic resin polymers and they are susceptible to change in the oral environment due to heat, humidity, constant forces, and saliva [35, 36]. The aligner material properties and deformation require more investigation in future studies to better simulate the clinical condition. Unfortunately, most of the aligner mechanical properties are patented by companies which cannot be disclosed to improve the FEM analysis [11]. Moreover, we will explore the stress distribution at TMJ area in future researches.

Furthermore, this is a static analysis which only revealed an initial movement tendency independent of the clinical dynamic process, as well as an alternate force for tooth movement. Multiple biological processes are involved in root resorption, including the root’s long-term contact with cortical bone [37]. Many factors, including patient cooperation, periodontal health, and root length can impact practical tooth movement. Therefore, the clinical translation of the conclusions should be taken with caution. In the future, more animal and clinical experiments should be carried out to acquire evidence at a higher level.

## Conclusions

In the present study, we biomechanically analyzed how the combination of clear aligners with Class II elastic affected the entire dentition during upper-molar sequential distalization. During this process, the anterior teeth exhibited labial inclination with a rotation center at the intersection of the apical and middle thirds of the roots. As the 1^st^ molar distalized, the loss of anterior anchorage worsened and the 2^nd^ molar exhibited a tendency for mesial palatal relapse. With the combination of Class II elastics, the anchorage of both anterior teeth and molars were effectively reinforced; nevertheless, the mandibular dentition with Class II elastics exhibited undesired movement. Compared with the use of buttons, the attachment of Class II elastics to aligners by precision cutting demonstrated superior anchorage control with reduced tooth displacement and corresponding stress on PDL and alveolar bone, which is a good alternative when proclination of upper incisors and extrusion of upper canines are unwanted.

## Supplementary Information

**Additional file 1.**

**Additional file 2.**

## Data Availability

The datasets used and analyzed during the current study are available from the corresponding author upon reasonable request.
