# Supplementary material for: Effects of upper-molar distalization using clear aligners in combination with Class II elastics: a three-dimensional finite element analysis
Source: BMC Oral Health. 2022 Dec 1;22:546. doi: 10.1186/s12903-022-02526-2 (PMC9714146; doi:10.1186/s12903-022-02526-2)
Supplement: Supplementary file 1 — Additional file 1. [file 12903_2022_2526_MOESM1_ESM.docx]

| Maxillary | Directions | x-axis | | | | | | y-axis | | | | | | z-axis | | | | | |
| --- | --- | --- | --- | --- | --- | --- | --- | --- | --- | --- | --- | --- | --- | --- | --- | --- | --- | --- | --- |
|  | Groups | Set I | | | Set II | | | Set I | | | Set II | | | Set I | | | Set II | | |
|  | Models | A | B | C | A | B | C | A | B | C | A | B | C | A | B | C | A | B | C |
| Central incisor | Crown | 0.0231 | 0.0191 | 0.0177 | 0.0263 | 0.0202 | 0.0197 | -0.1004 | -0.0869 | -0.0654 | -0.1113 | -0.0935 | -0.0718 | 0.0249 | 0.0111 | 0.0081 | 0.0261 | 0.0138 | 0.0087 |
|  | Root | -0.0035 | -0.0017 | -0.0007 | -0.0045 | -0.0026 | -0.0017 | 0.0312 | 0.0233 | 0.0199 | 0.0351 | 0.0264 | 0.0227 | -0.0250 | -0.0125 | -0.0093 | -0.0277 | -0.0152 | -0.0100 |
| Lateral incisor | Crown | 0.0020 | 0.0012 | 0.0008 | 0.0022 | 0.0013 | 0.0009 | -0.0976 | -0.0727 | -0.0549 | -0.1069 | -0.0740 | -0.0623 | 0.0254 | 0.0115 | 0.0071 | 0.0279 | 0.0135 | 0.0075 |
|  | Root | -0.0006 | -0.0004 | -0.0001 | -0.0006 | -0.0004 | -0.0001 | 0.0320 | 0.0246 | 0.0209 | 0.0358 | 0.0276 | 0.0234 | -0.0239 | -0.0112 | -0.0088 | -0.0276 | -0.0143 | -0.0098 |
| Canine | Crown | 0.0752 | 0.0382 | 0.0482 | 0.0779 | 0.0406 | 0.0521 | -0.0535 | -0.0295 | -0.0351 | -0.0522 | -0.0292 | -0.0366 | 0.0244 | -0.0144 | 0.0056 | 0.0259 | -0.0166 | 0.0078 |
|  | Root | -0.0229 | -0.0233 | -0.0223 | -0.0269 | -0.0272 | -0.0261 | 0.0232 | 0.0141 | 0.0161 | 0.0229 | 0.0142 | 0.0161 | -0.0234 | 0.0130 | -0.0053 | -0.0257 | 0.0157 | -0.0070 |
| First molar | Crown | 0.0635 | 0.0507 | 0.0326 | -0.1097 | -0.1611 | -0.1811 | -0.0293 | -0.0201 | -0.0185 | -0.0028 | -0.0016 | -0.0016 | 0.0441 | 0.0254 | 0.0171 | -0.0974 | -0.0873 | -0.0684 |
|  | Root | -0.0174 | -0.0113 | -0.0091 | 0.0418 | 0.0421 | 0.0421 | 0.0058 | 0.0040 | 0.0040 | 0.0101 | 0.0069 | 0.0050 | -0.0594 | -0.0433 | -0.0323 | 0.0947 | 0.0770 | 0.0709 |
| Second molar | Crown | -0.1347 | -0.1722 | -0.2223 | 0.0979 | 0.0673 | 0.0442 | -0.0255 | -0.0207 | -0.0178 | 0.0099 | 0.0066 | 0.0008 | -0.0885 | -0.0819 | -0.0620 | 0.0461 | 0.0358 | 0.0248 |
|  | Root | 0.0605 | 0.0568 | 0.0588 | -0.0273 | -0.0270 | -0.0270 | 0.0140 | 0.0097 | 0.0093 | -0.0072 | -0.0041 | -0.0037 | 0.0651 | 0.0597 | 0.0490 | -0.0348 | -0.0334 | -0.0303 |

**Supplementary file 1.** Three-dimensional displacement values for the maxillary anterior teeth and the molars (in mm).

The coordinate system was centered on each tooth( local coordinate system). A positive value on the x-axis represents the mesial surface of the teeth, a positive value on the y-axis represents the lingual surface of the teeth, and a positive direction on the z-axis represents towards the apex of the maxillary teeth.
