# Supplementary material for: Effects of upper-molar distalization using clear aligners in combination with Class II elastics: a three-dimensional finite element analysis
Source: BMC Oral Health. 2022 Dec 1;22:546. doi: 10.1186/s12903-022-02526-2 (PMC9714146; doi:10.1186/s12903-022-02526-2)
Supplement: Supplementary file 2 — Additional file 2. [file 12903_2022_2526_MOESM2_ESM.docx]

| Mandibular | Directions | x-axis | | | | y-axis | | | | z-axis | | | |
| --- | --- | --- | --- | --- | --- | --- | --- | --- | --- | --- | --- | --- | --- |
|  | Groups | Set I | | Set II | | Set I | | Set II | | Set I | | Set II | |
|  | Models | B | C | B | C | B | C | B | C | B | C | B | C |
| Central incisor | Crown | 0.0017 | 0.0019 | 0.0017 | 0.0020 | -0.0182 | -0.0188 | -0.0185 | -0.0195 | -0.0049 | -0.0052 | -0.0051 | -0.0052 |
|  | Root | -0.0014 | -0.0017 | -0.0014 | -0.0018 | 0.0058 | 0.0062 | 0.0060 | 0.0060 | 0.0012 | 0.0012 | 0.0013 | 0.0013 |
| Lateral incisor | Crown | 0.0048 | 0.0060 | 0.0052 | 0.0061 | -0.0217 | -0.0220 | -0.0220 | -0.0223 | -0.0049 | -0.0060 | -0.0056 | -0.0063 |
|  | Root | -0.0060 | -0.0074 | -0.0061 | -0.0079 | 0.0074 | 0.0074 | 0.0075 | 0.0076 | 0.0033 | 0.0044 | 0.0041 | 0.0043 |
| Canine | Crown | 0.0143 | 0.0162 | 0.0154 | 0.0165 | -0.0077 | -0.0078 | -0.0078 | -0.0079 | -0.0031 | -0.0033 | -0.0033 | -0.0035 |
|  | Root | -0.0169 | -0.0182 | -0.0176 | -0.0199 | 0.0050 | 0.0061 | 0.0060 | 0.0070 | 0.0029 | 0.0034 | 0.0030 | 0.0037 |
| First molar | Crown | 0.0147 | 0.0151 | 0.0151 | 0.0155 | 0.0090 | 0.0096 | 0.0102 | 0.0099 | 0.0109 | 0.0114 | 0.0108 | 0.0124 |
|  | Root | 0.0010 | 0.0008 | 0.0006 | 0.0004 | -0.0099 | -0.0120 | -0.0102 | -0.0132 | 0.0032 | 0.0030 | 0.0032 | 0.0030 |
| Second molar | Crown | 0.0100 | 0.0101 | 0.0102 | 0.0103 | 0.0030 | 0.0032 | 0.0032 | 0.0033 | 0.0021 | 0.0028 | 0.0027 | 0.0028 |
|  | Root | -0.0029 | -0.0029 | -0.0030 | -0.0031 | -0.0018 | -0.0022 | -0.0020 | -0.0026 | -0.0052 | -0.0057 | -0.0055 | -0.0056 |

**Supplementary file 2.** Three-dimensional displacement values for the mandibular anterior teeth and the molars (in mm).

The coordinate system is centered on each tooth( local coordinate system). A positive value on the x-axis represents the mesial surface of the teeth, a positive value on the y-axis represents the lingual surface of the teeth, and the z-axis represents a positive direction towards the incisor/occlusal part of mandibular teeth.
